# Necrotizing Pneumonia as a Complication of Community-Acquired Pneumonia in Adults at a Tertiary Institution

**DOI:** 10.3390/jcm14124086

**Published:** 2025-06-10

**Authors:** Leela Krishna Teja Boppana, Samantha Isern, Kaitlyn N. Romero, Jason Ferreira, Gerard Garvan, Tracy Ashby

**Affiliations:** 1 Division of Pulmonary and Critical Care Medicine, University of Florida—Jacksonville, 655 W 8th Street, Jacksonville, FL 32209, USA; ferreiragator@gmail.com (J.F.); tracy.ashby@jax.ufl.edu (T.A.); 2 Division of Internal Medicine, University of Florida—Jacksonville, Jacksonville, FL 32209, USA; samantha.isern@jax.ufl.edu (S.I.); kaitlyn.romero@jax.ufl.edu (K.N.R.); 3 UF Center for Data Solutions, University of Florida—Jacksonville, Jacksonville, FL 32209, USA; gerard.garvan@jax.ufl.edu

**Keywords:** necrotizing pneumonia, community-acquired pneumonia, pulmonary gangrene, mechanical ventilation

## Abstract

**Background/Objectives**: Necrotizing pneumonia (NP) is an uncommon, severe complication of community-acquired pneumonia (CAP) associated with increased hospital length of stay and high morbidity and mortality. Although this entity was described several decades ago, there is no consensus on radiological criteria for diagnosis, optimal antibiotic duration, or data on clinical outcomes in adults. Given the paucity of data on this entity, a retrospective cohort study was conducted at our institution to evaluate factors associated with all-cause mortality, hospital length of stay, and duration of antibiotics. **Methods**: An IRB-approved retrospective cohort analysis was conducted through electronic health record review at a tertiary academic center at the University of Florida—Jacksonville. The electronic health record was queried for a list of all hospitalizations from 1 January 2016 to 31 December 2023 with an International Classification of Diseases, 10th revision diagnosis code of J85.0 (gangrene and necrosis of the lung). The primary outcome was all-cause mortality, and secondary outcomes were hospital length of stay and duration of antibiotics. **Results**: A total of 57 patients met the definition of necrotizing pneumonia and were included in our study. Fourteen (24.6%) patients died while hospitalized. The mean length of hospital stay was 26.6 days, and the median duration of antibiotics was 28 days. The only statistically significant predictor in the model of all-cause mortality was the requirement of mechanical ventilation, with mortality being 27 times more likely in patients requiring mechanical ventilation (OR 27.6 (95% CI (2.6924, 671.9648)); *p* = 0.011). **Conclusions**: To our knowledge, this is the largest cohort of adult patients with NP in the literature. We found that mortality was 24.6%, with the requirement of mechanical ventilation associated with 27 times higher risk of mortality on multivariable logistic regression analysis.

## 1. Introduction

Necrotizing pneumonia (NP) is an uncommon, severe complication of community-acquired pneumonia (CAP) [1] associated with increased hospital length of stay and high morbidity and mortality [2]. This condition was first described in the 1940s in adults, with the incidence of NP being less than 1%, but exact numbers are not known [3]. NP lies on a spectrum of disease between lung abscess and pulmonary gangrene. It is characterized by pulmonary inflammation with consolidation, peripheral necrosis, and multiple small cavities due to compromise of the bronchial and pulmonary vasculature, which favors uncontrolled bacterial replication [1,3] and impairment in antibiotic delivery [3]. The exact mortality is unknown and is variable from 8% to 45% [4,5] in the literature.

Although NP was described several decades ago, there is no consensus on radiological criteria for diagnosis, optimal antibiotic duration, or data on clinical outcomes in adults. The initial presentation is often indistinguishable from CAP, and NP is suspected in patients admitted with CAP who fail to improve or clinically deteriorate after at least 72 h of appropriate antibiotic therapy [6]. When suspected, the diagnosis is made using computed tomography (CT) of the chest, which shows loss of normal pulmonary parenchymal architecture and areas of decreased parenchymal enhancement, representing liquefaction, that are replaced by multiple small or fluid-filled cavities [7]. NP can progress to complications [8], which include septic shock, bronchopleural fistula, and empyema, among others.

The current mainstay [5] of treatment is aggressive medical management with antibiotic therapy and drainage of complicated parapneumonic effusion (CPPE)/empyema. Current treatment of NP is based on international society guidelines for CAP [9,10] and initiating empiric therapy based on an individual’s risk of community- or hospital-acquired organisms and additional patient-specific risk factors. Isolating a causative pathogen is a crucial step in the treatment of NP, as it allows tailoring antibiotic therapy and better outcomes [3]. The duration of antibiotic therapy in adults is not well known and has been extrapolated from the pediatric literature, where the usual duration is 2 to 4 weeks [6,11]. Historically, surgery of devitalized tissue was extensively reported [1,3,5,8,12] for NP with previous indications for surgery including failure of medical management, persistent or significant hemoptysis, or evidence of extensive gangrene. The optimal timing and indication for surgical interventions are not well established, and this remains a highly debated topic [5].

Currently, there are no guidelines for the management of NP, and the available literature consists of case series and small retrospective studies describing various treatment approaches in adults [1,4,8]. Overall, studies on NP focus on infections in the pediatric population [6,7,13,14,15]. Given the paucity of data on this entity in adults, a retrospective cohort study was conducted at an urban tertiary healthcare center, which serves a large uninsured patient population. The primary outcome was all-cause mortality, and secondary outcomes were hospital length of stay and duration of antibiotics.

## 2. Materials and Methods

### 2.1. Study Design and Setting

We performed a retrospective cohort analysis through chart review at a tertiary academic center, the University of Florida in Jacksonville, Florida. The electronic health record (EPIC©) was queried for patients from 1 January 2016 to 31 December 2023 hospitalized with an International Classification of Diseases, 10th revision (ICD 10) diagnosis code of J85.0 (Gangrene and necrosis of the lung) who had an initial admission diagnosis of CAP.

This study was conducted in accordance with the Declaration of Helsinki (as revised in 2013). Our study received institutional review board (IRB) approval, and patient consent for this retrospective study was waived.

The primary outcome was all-cause mortality. Secondary outcomes included hospital length of stay and duration of antibiotics. The duration of antibiotics included the total number of days a patient received antibiotics while admitted and after discharge, if applicable. This included the initial empiric regimen chosen as per the treating physician based on patient-specific risk factors, subsequent modifications by the treatment team based on microbiological data while admitted, and the anticipated planned duration at the time of discharge. In the case of death, the number of days of antibiotics leading up to the day of death was the duration used. Length of hospital stay was counted from the day of admission to the day of discharge (whether home, facility, or death).

### 2.2. Participants

Given the lack of a consensus definition for NP, a definition proposed in a recent article was adapted for our study [4]. NP was defined as “one necrotizing cavity involving 50% or more of a lobe, or at least 2 or more smaller multilobar cavities on cross-sectional imaging (CT) scan of the chest.”

**Figure 1:**
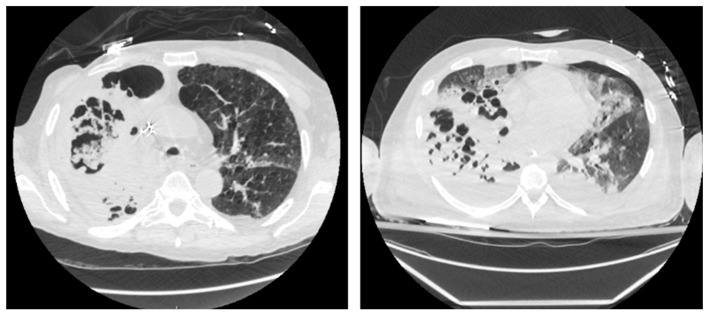
Cross-sectional images of 2 patients from our cohort with necrotizing pneumonia.

Cross-sectional imaging was individually reviewed by 2 of 3 physicians (LKTB, SI, or KR) to ensure they met the agreed-upon definition of necrotizing pneumonia. This was carried out in a blinded fashion without reviewing clinical characteristics or outcomes. In the event of a discrepancy, a 3rd independent review determined inclusion to limit selection bias. Cross-sectional images from 2 patients with NP from our cohort are shown (Figure 1).

Patients were included if they were hospitalized with an ICD 10 code J85.0, were at least 18 years of age or older, and met the above-described definition for NP. Patients were excluded in the event they were diagnosed with lung cancer but not started on treatment or were currently being treated for lung cancer.

### 2.3. Variables

We collected baseline demographics, initial vital signs on presentation to the emergency department, duration of symptoms prior to presentation, hospital length of stay, ICU length of stay (if any), mode of oxygenation (invasive (mechanical ventilation) and non-invasive (nasal cannula, high-flow nasal cannula, and positive airway pressure (PAP) therapy)) at time of hospital admission, and need for vasopressors.

Duration of mechanical ventilation was calculated as the total number of days from the date of endotracheal intubation to the date when the patient was liberated from mechanical ventilation. We collected microbiologic data, including respiratory culture growth, total duration of antibiotics, interventions related to NP (including but not limited to chest tube placement, debridement, wedge resection, lobectomy, and pleurodesis), and discharge status (including death during the admission of the NP diagnosis).

### 2.4. Quantitative Variables and Statistical Analysis

Data were checked for missingness and distributional form. We conducted a complete case analysis, including only those patients with available data for all variables relevant to each specific analysis. Groups (survived vs. died) were compared on the following variables: age, smoking history, presence of comorbidity, extent of pneumonia, respiratory culture growth (sputum, endotracheal aspirate, or BAL), monomicrobial vs. polymicrobial growth, methicillin-resistant staphylococcus aureus (MRSA) growth, need for mechanical ventilation during hospital stay, septic shock, and inpatient surgery. The Wilcoxon rank sum test [16] was used to compare mortality on the numeric variable age. The chi-squared test was used on the categorical variables MRSA and mortality. Fisher’s exact tests were used for all other variables. Multivariable binary logistic regression [17] was conducted with in-hospital mortality (dead vs. survived) as the dependent outcome to simultaneously examine the effect size of predictor variables (need for mechanical ventilation and septic shock). The level of significance was set at 0.05. R was used for all analyses and graphing (R Core Team 4.4.0) [18].

## 3. Results

**Figure 2:**
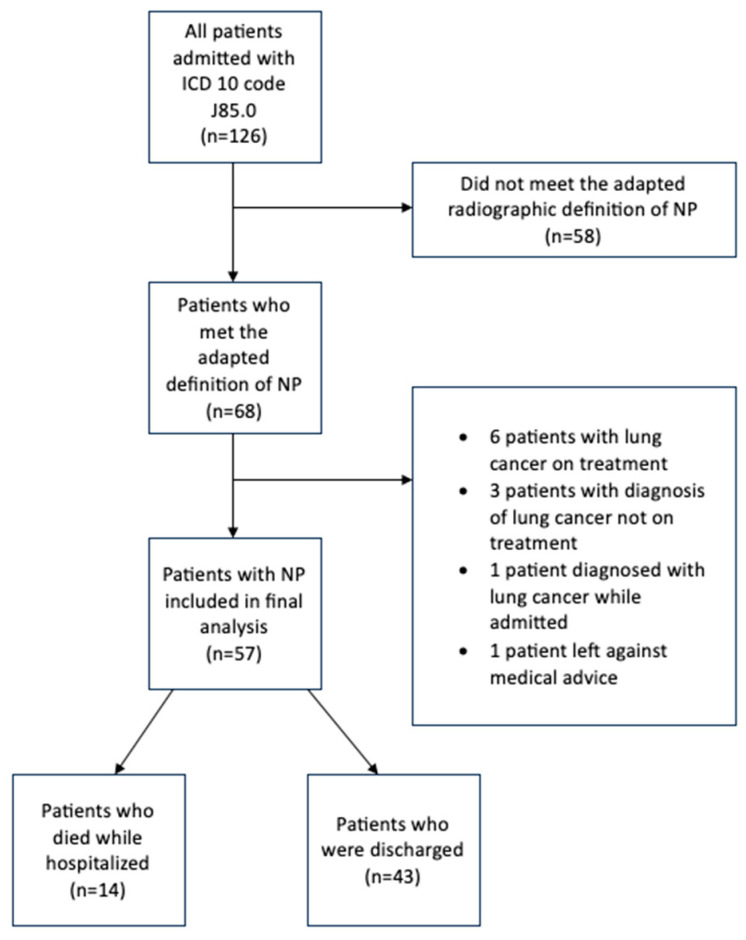
Study flow chart.

Of 126 patients admitted with an ICD 10 code J85.0, 57 were included in the final analysis. The most common reason for exclusion was not meeting the adapted definition for NP (Figure 2). Patients included in this study had a mean age of 55 and were commonly Caucasian males (Table 1), with the age range being 19 to 83 years. A majority of patients were current or active smokers, and 21 patients had a spirometry diagnosis of chronic obstructive pulmonary disease (COPD). Patients had onset of symptoms usually within one week prior to presentation, and the median mean arterial pressure (MAP) on presentation was 85.0 mmHg [72.0, 98.0].

CT imaging revealed multifocal bilateral necrotizing pneumonia in 21 (36.8%) patients (Table 2). Thirteen patients had a concomitant complicated parapneumonic effusion or empyema by physician diagnosis, and five (8.8%) patients developed bronchopleural fistula during their hospital course. Sixteen (28.1%) patients had a chest tube placed during their hospital course, of which eleven patients initially had chest tube placement for complicated parapneumonic effusion or empyema and five patients for bronchopleural fistula/pneumothorax.

For those patients with microbiological findings, six patients had a positive viral polymerase chain reaction test. Of these six patients, two patients tested positive for rhinovirus and one each for influenza A, influenza B, and SARS-CoV-2. Microbiologic culture growth was noted in 46 patients. Of patients with positive culture results, 20 (35.1%) patients had monomicrobial growth and 26 (42.3%) had polymicrobial growth. MRSA (N = 12) was the most common bacteria isolated overall. Other bacteria isolated included *Pseudomonas* sp. (N = 5), MSSA (N = 4), *Escherichia coli* (N = 3), and other Gram-negative rods (N = 15) (Table 3).

From the emergency department, 27 patients required direct ICU admission. Twenty patients needed mechanical ventilation during their hospital course, and sixteen patients needed vasopressors for septic shock. The mean length of hospital stay was 26.6 days. For the 20 patients who required invasive mechanical ventilation, the mean time on the ventilator was 8.5 days. The median duration of antibiotics was 28 days. Eight patients underwent VATS with decortication for complicated pleural effusion and/or empyema. One patient had intraoperative debridement performed at the time of VATS. Fourteen (24.6%) patients died while hospitalized (Table 4).

In an attempt to determine which patient attributes are associated with all-cause mortality, bivariate tests were utilized on the variables age, smoking history, co-morbidities, extent of pneumonia (single lobe, multifocal ipsilateral, or multifocal bilateral involvement), respiratory culture growth (monomicrobial vs. polymicrobial), MRSA growth, need for mechanical ventilation during hospital stay, septic shock, and need for inpatient surgery. Septic shock and the need for mechanical ventilation were found to be significant (Table 5). Then, multivariable logistic regression analysis (Table 6) was performed, and the only statistically significant predictor in the model of all-cause mortality was the requirement of mechanical ventilation, with mortality being 27 times more likely to occur in patients requiring mechanical ventilation (OR 27.6 (95% CI (2.6924, 671.9648)); *p* = 0.011).

## 4. Discussion

To our knowledge, this is the largest study of adult patients with necrotizing pneumonia in the literature. Patients with necrotizing pneumonia in our cohort had a mortality of 24.6%, with the most significant risk factor for mortality being the need for mechanical ventilation. The median duration of antibiotic therapy was 28 days, and the mean length of hospital stay was 26.6 days, which highlights the substantial resource utilization for this disease entity.

In the current study, the demographics of patients admitted with NP are similar to other case series and cohorts in the literature [1,4,8,19]. Patients are likely to be middle-aged males with comorbid conditions including active cigarette smoking, COPD, alcohol use, HIV, hypertension, and diabetes. While not all-inclusive, these observed comorbidities are well-established risk factors for CAP [20,21], as well as NP, which is a severe form or progression of CAP [3,6,7].

Among patients with respiratory culture growth, a majority of patients had polymicrobial growth. The microbiological findings were more commonly consistent with hospital-acquired organisms than those that would be typically seen in a community-acquired setting, with a predominance of Gram-negative rods (*Pseudomonas* species, *Escherichia coli*, and others), followed by MRSA. While we did not have data available for history of prior hospitalizations, it is notable that 37% of patients in our cohort had spirometric evidence of COPD. In such patients, Staphylococcus Aureus and Gram-negative bacilli are common causes of CAP in addition to typical CAP organisms, which could be a possible explanation for the microbiological growth [9]. Additionally, given the varying intervals into the hospitalization that cultures were obtained, one cannot rule out that organisms could have been acquired while hospitalized and may not actually represent the initial organism that caused NP. Similarly, in the cohort by Larose et al. [4], monomicrobial growth was common, but the organisms were also not typical community-acquired organisms. In another case series [8], monomicrobial growth was common, with *Klebsiella pneumoniae* being a common isolate. There are more data on NP in children, and the common microbes in that age group are *Streptococcus pneumoniae*, *Staphylococcus aureus*, and *Mycoplasma pneumoniae* [13], which are organisms more commonly seen in a community-acquired setting. Overall, this shows the importance of obtaining respiratory sampling so antibiotic therapy can be adequately tailored to the organism isolated based on identification and sensitivity. *Staphylococcus aureus* remains a common pathogen in NP [2,3,22,23,24], with the significance of this being its ability to produce Panton–Valentine leukocidin (PVL) cytotoxin [24], which has the potential to cause severe and fatal necrotizing pneumonia in young, immunocompetent adults. In our cohort, monomicrobial or polymicrobial growth and MRSA isolation did not have an influence on outcomes.

With the complexity and spectrum of disease associated with NP, the duration of antibiotic therapy for NP has not been defined, and current international guidelines on CAP do not provide guidance on this [9,10]. Given the severity of the illness, many practitioners opt for longer durations. In children, Masters et al. [6] found a median antibiotic course duration of 28 days across their systematic review of three case series [7,14,15]. Our cohort has a similar median antibiotic duration of 28 days, with treatment durations extending up to 42 days at the discharging physician’s discretion. For patients who died, we analyzed microbiological growth and antibiotics administered during the hospital course. In two patients who died, the initial antibiotic regimen chosen for CAP coverage did not cover the organism (MRSA), which was isolated at approximately 48 and 72 h after admission, and therefore, there was a delay in starting appropriate antibiotic treatment. This signifies the importance of selecting an initial empiric antibiotic regimen based on patient specific risk factors. For nine other patients who died and had microbiological growth, the initial regimen selected provided appropriate coverage.

Historically, in patients who were not improving despite medical therapy with antibiotics, surgery was employed. In our institution, surgery was performed on eight patients, with VATS/decortication being performed for CPPE/empyema and one patient having local debridement concurrently at the time of VATS. Several case series [1,8,12,25] describe surgery (wedge resection and lobectomy) being performed for necrotizing pneumonia and pulmonary gangrene with varying success, but the selection criteria are not clearly defined. Surgery was historically indicated [1] to manage those who fail medical management (uncontrolled sepsis despite medical therapy), have massive hemoptysis, develop pulmonary gangrene, or lack alternative treatment options. The approach to surgery in our institution is similar to the experience of Larose et al., where surgical decortication was employed for CPPE/empyema. The question still remains if there could be a role for lung resection in certain patients who fail to improve despite appropriately targeted antimicrobial therapy, but the current direction favors antimicrobial targeted antibiotic therapy and surgery utilized for treatment of CPPE/empyema [5]. In cases where surgery is being considered, a multidisciplinary approach including consultation with a thoracic surgeon may be beneficial, as described in the institutional experience by Larose et al., but is not standard of care as previously described.

Our cohort had a mortality of 24.6%, which is higher than other cohorts. However, this is within the range (8% to 40%) reported in other studies [1,8,12,25,26,27,28]. Reasons for this variability in mortality include small case series [1], case series with patients who were only surgically managed for NP [1,12], and a heterogenous patient population [1,12,26] that included patients with lung abscess and/or pulmonary gangrene. In our cohort, the mortality could be due to patient comorbidities, acuity, nosocomial microbiology, and potentially a lack of standardization in management, as our institution did not utilize a multidisciplinary team approach as described by Larose et al. Additionally, while we did not record scores or obtain data for risk prediction models (Pneumonia Severity Index, APACHE II, or Charlson Comorbidity Index), our cohort was critically ill, as shown by the number of patients who were in septic shock, needed mechanical ventilation, and were directly admitted to the ICU from the emergency department. The mean hospital length of stay was 26.6 days, consistent with other studies in the literature [4,29].

Finally, an analysis of characteristics for all-cause mortality found that the need for mechanical ventilation was statistically significant, with a 27 times increased likelihood for mortality. However, the wide confidence interval suggests a variability in patient population. Evidence of this variability is suggested by the mean number of days (SD) on mechanical ventilation and hospital length of stay, with one patient in our cohort having spent 161 days on mechanical ventilation and eventually dying during his hospital course. Additionally, patients with NP are heterogeneous due to factors including but not limited to extremes of age and comorbidities, which contributes to this large variability, and thus interpretation should proceed with caution. Studies have shown that the need for mechanical ventilation [5,29,30] in patients with CAP is associated with a high mortality due to the extent of lung injury and thus is a representation of the severity of illness. Although 16 patients (28.1%) had septic shock, we found that this was not associated with all-cause mortality in our cohort. Finally, while the extent of pneumonia at the time of diagnosis of NP was recorded, radiological data towards the end of the hospital course were not evaluated for progression of initial findings or development of other organ failure, which may have contributed to their mortality.

## 5. Limitations

Several limitations of the present study should be recognized. Although at least two independent physicians individually reviewed all CT imaging, chest radiographs of patients were not reviewed as they did not meet our adapted definition of NP. Therefore, patients diagnosed with NP based on a chest radiograph would have been excluded from our cohort, raising the possibility of selection bias. Patients with NP who were not coded with an ICD-10 code J85.0 would also not have been reviewed. Additionally, there is a possibility of anchoring bias, as we would have assumed all patients being screened were diagnosed with NP. However, a patient with undiagnosed lung cancer could be misdiagnosed with NP, as there is an overlap in radiographic features between these two disease processes. We acknowledge that decisions regarding treatment and overall management depend on the treating physician/team, and not all patients may have had a similar treatment course, including selection of initial empiric antibiotics, which may have affected outcomes. Disease severity scores were not calculated to objectively determine illness severity in our study because many patients did not have an arterial blood gas at the time of evaluation in the emergency department, with pH and/or PaO_2_ needed for several of these scores (APACHE II, SAPS II, SOFA score, and PSI). The findings also support a widely heterogeneous population, and a number of key contributors to outcomes may not have been collected or, for that matter, discovered at the time of this study. Given the retrospective study design and sample size, we prioritized logistic regression for interpretability and clinical relevance but acknowledge the potential of alternative methods that could be used for statistical analysis. The strengths of this study include the largest cohort currently in the literature and patient selection at a large tertiary care hospital.

## 6. Conclusions

To our knowledge, this is the largest cohort of adult patients with NP in the literature. In our cohort, mortality was 24.6%, with the requirement of mechanical ventilation associated with 27 times higher risk of mortality on multivariable logistic regression analysis. The mean length of hospital stay was 26.6 days, and the median duration of antibiotic therapy was 28 days. Utilizing an institutional multidisciplinary team approach in patients with NP could potentially lead to better patient outcomes, and this could be an area of future research.

## Figures and Tables

**Table 1:** Baseline characteristics of patients with necrotizing pneumonia.

	N = 57
**Age**	
Mean	55.0 (15.0)
Median	57.0 [43.0, 66.0]
**Male**	31 (54.4%)
**Race**	
White	29 (50.9%)
Black or African American	25 (43.9%)
Asian	1 (1.8%)
Other	2 (3.5%)
**Co-Morbidities**	
Smoker	47 (82.5%)
Alcohol Use	10 (17.5%)
Diabetes	12 (21.1%)
Hypertension	27 (47.4%)
Chronic kidney disease	1 (1.8%)
Chronic obstructive pulmonary disease	21 (36.8%)
HIV	7 (12.3%)
History of malignancy	9 (15.8%)
**Need for mechanical ventilation**	20 (35.1%)
**Septic shock needing vasopressors**	16 (28.1%)

Variables reported as medians with interquartile range [IQR] or mean with standard deviation (SD) as appropriate.

**Table 2:** Clinical and radiographic data of patients with necrotizing pneumonia.

	N = 57
**Onset of Symptoms**	
<1 week	35 (61.4%)
1–2 week	11 (19.3%)
>2 weeks	11 (19.3%)
**MAP on admission**	
Mean	86.6 (20.4)
Median	85.0 [72.0, 98.0]
**Extent of Pneumonia**	
Single lobe	19 (33.3%)
Multifocal ipsilateral	17 (29.8%)
Multifocal bilateral	21 (36.8%)
**Pneumonia-associated complications**	
Septic shock	16 (28.1%)
Complicated parapneumonic effusion/empyema	13 (22.8)
Bronchopleural fistula	5 (8.8%)
**Need for chest tube placement**	16 (28.1%)

Variables are reported as medians with interquartile range [IQR] or mean with standard deviation (SD) as appropriate.

**Table 3:** Microbiological data of patients with necrotizing pneumonia.

Characteristics	N = 57
**Viral PCR positivity**	
Influenza A	1 (1.8%)
Influenza B	1 (1.8%)
Rhinovirus	2 (3.5%)
SARS-CoV-2	1 (1.8%)
**Respiratory culture (sputum, tracheal aspirate, or bronchoalveolar lavage) growth**	
Monomicrobial	20 (35.1%)
Polymicrobial	26 (61.4%)
None identified	11 (19.3%)
**Associated pathogens**	
*Methicillin-sensitive Staphylococcus Aureus* (MSSA)	4
*Methicillin-resistant Staphylococcus Aureus* (MRSA)	12
*Pseudomonas* sp.	5
*Escherichia coli*	3
*Streptococcus pneumoniae*	2
*Streptococcus pyogenes*	2
Other Gram-negative rods	15
Mixed flora	12

**Table 4:** Interventions and outcomes of patients with necrotizing pneumonia.

Characteristics	N = 57
**Hospital length of stay (days)**	
Mean	26.6 (29.2)
Median	16.0 [12.0, 29.0]
**ICU admission from emergency department**	27 (47.4%)
**Time spent on mechanical ventilation (days), mean**	8.54 (24.0)
**Time spent on mechanical ventilation (days), median**	0 [0, 6]
**Duration of antibiotics (days), median**	28 [21–42]
**Surgery performed**	**8**
Video-assisted thoracoscopic surgery (VATS) with decortication	8
Local debridement	1
**Mortality**	14 (24.6%)

Variables reported as medians with interquartile range [IQR] or mean with standard deviation (SD) as appropriate.

**Table 5:** An analysis of characteristics for in-hospital mortality of patients with necrotizing pneumonia.

	Yes (N = 14)	No (N = 43)	*p*-Value
**Age**			
Mean (SD)	57.7 (14.4)	54.1 (15.3)	0.458 *
**Smoking history**			
Yes	10 (71.4%)	37 (86.0%)	0.24
No	4 (28.6%)	6 (14.0%)	
**Presence of comorbidity**			
No	2 (14.3%)	5 (11.6%)	1
Yes	12 (85.7%)	38 (88.4%)	
**Extent of pneumonia**			
Single lobe	3 (21.4%)	16 (37.2%)	0.588
Multifocal ipsilateral	5 (35.7%)	12 (27.9%)	
Multifocal bilateral	6 (42.9%)	15 (34.9%)	
**Respiratory culture growth (sputum/tracheal aspirate or BAL)**			
No	2 (14.3%)	9 (20.9%)	0.714
Yes	12 (85.7%)	34 (79.1%)	
**Monomicrobial vs. polymicrobial growth on respiratory culture**			
None	2 (14.3%)	9 (20.9%)	0.664
Monomicrobial	4 (28.6%)	16 (37.2%)	
Polymicrobial	8 (57.1%)	18 (41.9%)	
**MRSA**			
No	8 (57.1%)	27 (62.8%)	0.471 ^†^
Yes	4 (28.6%)	8 (18.6%)	
**Need for mechanical ventilation during hospital stay**			
Yes	13 (92.9%)	7 (16.3%)	<0.001
No	1 (7.1%)	36 (83.7%)	
**Septic shock**			
Yes	3 (21.4%)	38 (88.4%)	<0.001
No	11 (78.6%)	5 (11.6%)	
**Bronchopleural fistula**	12 (85.7%)	40 (93.0%)	0.587
Yes	2 (14.3%)	3 (7.0%)	
No			
**Complicated parapneumonic effusion/empyema**			
Yes	10 (71.4%)	34 (79.1%)	0.715
No	4 (28.6%)	9 (20.9%)	
**Inpatient surgery**			
Yes	2 (14.3%)	6 (14.0%)	1
No	12 (85.7%)	36 (83.7%)	

* For this variable we performed the Wilcoxon rank sum test. ^†^ For this variable we performed the chi-squared test, and for all other variables we performed the Fisher exact test.

**Table 6:** Multivariable linear regression analysis of in-hospital mortality.

	Coefficient	exp(Coefficient)	95% CI for Coefficient	95% CI for exp(Coefficient)	*p*-Value
**Need for mechanical ventilation**	3.32	27.60	(0.99, 6.51)	(2.69, 671.96)	0.011
**Septic shock**	1.31	0.04	(0.74, 3.43)	(0.48, 30.82)	0.203

## Data Availability

Data can be made available based on reasonable requests to the authors.

## Abbreviations

The following abbreviations are used in this manuscript:

BAL	Bronchoalveolar lavage
CAP	Community-acquired pneumonia
CPPE	Complicated parapneumonic effusion
CT	Computed tomography
ICU	Intensive care unit
MRSA	Methicillin-resistant Staphylococcus Aureus
NP	Necrotizing pneumonia
VATS	Video-assisted thoracoscopic surgery
